# An open-source biaxial tensile tester with automated pre-tensioning for mechanical studies of canvas paintings

**DOI:** 10.1016/j.ohx.2023.e00412

**Published:** 2023-02-28

**Authors:** Antonio Iaccarino Idelson, Miguel Sánchez López, Roger Groves

**Affiliations:** a Delft University of Technology, Kluyverweg 1, 2600 GB Delft, the Netherlands; b Universitat Politècnica de València, Camino de Vera, s/n. 46022 – Valencia, Spain

**Keywords:** Biaxial tester, Canvas paintings, Pre-tensioning, Mechanics of paintings

## Abstract

The mechanical aspects of canvas painting conservation and the study of the effects of conservation treatments benefit greatly from quantifying the mechanical characteristics of the materials. However, this is seldom possible as only few labs have the necessary equipment. This paper presents the development of a biaxial tester to be used for samples of canvas paintings which exhibit orthotropic behavior under biaxial loads. The machine was built as the first step of ongoing Ph.D. research on the mechanics of canvas paintings. An effort was made to create a system that is easy to assemble, with parts that are easy to source and with an overall cost well below the commercial units available. The control software includes the function of automated pre-tensioning to improve the accuracy of the measurement. Our broader purpose here is to make an easy-to-replicate machine available to help conservators and conservation scientists perform tensile tests to make informed choices in materials science.

## Graphical abstract

**Figure:**
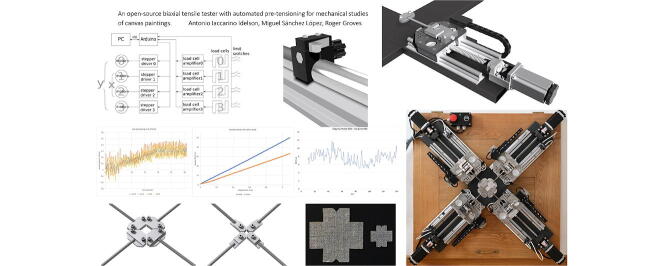

Specifications table.

**Table:** 

Hardware name	Biaxial tester for canvas paintings samples
Subject area	• Engineering and materials science • Educational tools and open-source alternatives to existing infrastructure • Canvas paintings conservation
Hardware type	• Other: Material testing system
Closest commercial analog	• No commercial analog is available
Open-source license	Creative Commons Attribution 4.0 International
Cost of hardware	• Approximate cost: € 2500
Source file repository	https://zenodo.org/record/7541996#.Y8Wd1uLMIqs

## Hardware in context

A typical painter’s canvas consists of yarns woven together at a 90-degree angle, the warp and weft, and it exhibits different mechanical properties depending on the direction being stressed. If the two are stressed simultaneously, the resulting force will still be different because each direction influences the behavior of the other. Researchers have been studying this relationship for decades, first in the textile industry [1], then in the conservation of paintings on canvas [2].

In standard conditions, a painting on its stretcher is subjected to tensions simultaneously acting in two orthogonal directions in the painting’s plane [[3], [4]]. Current research is mainly based on uniaxial tests performed on each of the main directions of the canvas, but uniaxial data can, in some cases, become misleading. The role of biaxial testing is to provide data on the behaviour of a painting and to read the interaction between the orthogonal forces. Only in recent years have biaxial testing machines been built by leading industries for specific engineering purposes^1^, and some low-cost machines have been designed with the use of Arduino boards [[5], [6]]. Biaxial testing machines need to be designed to meet the needs of a specific field of investigation regarding loads, sample dimensions and clamping. Currently available machines are typically targeted for architectural tension structures based on coated textiles [[7], [8]] or for biomedical purposes [9] and are out of scale for painting conservation, being either too large and powerful or miniaturized. An assessment of the history of the machines used for testing the mechanical behavior of paintings on canvas up to the end of the 20th century was carried out extensively by C. Young [10]. Among the historical biaxial testing machines, we only refer here to that built in 1963 by Clulow and Taylor for the textile industry [1]. It was designed to apply forces simultaneously on the four ends of the two axes, thus allowing to keep the center of the sample in a stable position. Force values were read through springs located at one end of each axis. The machine could test cruciform samples of 10 × 10 cm, but 10 cm uniaxial arms of the same material at each end were needed to redistribute the non-orthogonal stresses. Therefore, the actual sample size was 30 × 30 cm.

The first biaxial testing machine for canvas painting conservation was built in 1982 by Russel and Berger [2] and implemented in 1986 [11]. It was designed for testing relatively large samples (25 × 25 cm), monitoring tensions over long-duration tests, and it also intended to simulate environmental changes. Force was actuated at one end of each axis with manual adjustment bolts, and a micrometer screw allowed fine adjustment at the opposite end. The motion was not symmetric, and the sample was not kept centered in the setup during testing. Samples were connected with fiberglass threads glued with epoxy resin dot joints to the moving bars of the machine. Such a fixture method allowed lateral movements, favoring tension redistribution within the sample. Tension was applied by turning the nuts and reading with two load cells placed in the moving ends. In the first version [2], tension was applied with dead weights and pulleys. The device has been active over a short time span in the mid-1980 s [[2], [11]].

The second biaxial tester in conservation was built at the Tate Gallery for C. Young's Ph.D. in 1996 [10]. Data was presented in 1996 [12] and in 1999 [13]. The typical sample dimension was 26 × 20 cm. At one end of each axis, a stepper motor and a load cell were placed on coaxial rail guides; at the opposite end was a manual adjustment stage. Like in the Russell-Berger, motion was asymmetric from the center; still, displacements and strains were measured using optical Electronic Speckle Pattern Interferometry (ESPI) [14], a technique gathering information on the entire sample. Stepper motors were used for fine adjustment of position and force and to operate and measure displacement when performing “load-extension” (stress–strain) tests. The tester has been active since 1996 [[10], [12]] and was still in use at the Courtauld Institute in London in 2006^2^, when the machine had been modified to meet research needs.

Machines built after 1999 in the field are few. One was built for Chiriboga's Ph.D. [15] at the Delft University of Technology, Faculty of Aerospace Engineering, in 2013. Chiriboga's, like the previously described machines, was intended to study large samples (40 × 40 cm or 30 × 40 cm). A very simple structure, with hand-operated screws actuators and a load cell on each axis, it was used to set a known value of tension before exciting the sample with a sound wave for reading vibration and deformation patterns with a laser vibrometer. A similar stretching device was used by Leila Sauvage to study vibrations in pastels on paper [16]. The latest use of a biaxial testing machine in conservation to our knowledge (2020), is used to evaluate the mechanical performances of adhesives used for butt joining individual threads in canvas paintings tear mending [17]. The machine works with four motors, thus with a symmetric motion from the center, and its most interesting feature seems to be that it allows a finely adjustable pre-tension [18], though this is not thoroughly described. A similar machine was presented in 2019 by the same working group at Saarland University [19].

Our aim is to create a new biaxial tensile test machine that is as inexpensive and easy to build as possible, in order to make it available to the largest number of researchers, obtaining high-quality results. The machine presented in this paper is the fruit of the interdisciplinary work and collaboration between a painting conservator, a computer scientist and an expert in materials characterization. Biaxial tensile tests procedures for canvas using a cruciform sample are currently being developed by ISO^3^, under the name: ISO/AWI 13,118 “Textile - Biaxial tensile properties of a woven fabric - Determination of elasticity properties using a cruciform test piece”.

In the following sections of this paper we shall start with the definition of the test conditions implying requirements for the machine design. Hardware will be described, along with the firmware and the operating software. A step-by-step assembling guide and operating instructions will follow, concluding with a description of the results of some of the tests possible with the machine.

## Hardware description

### General requirements for tensile testing of canvas paintings samples

The structure of a canvas painting is generally described by the canvas support, woven with natural or synthetic fibers; a “ size” layer, with animal glue or a synthetic emulsion, making the canvas surface less absorbent and more suitable to receive subsequent layers; a thicker “preparation” layer, with binders and charges, filling the spaces between the threads and building the surface that will receive the paint [20]; finally, the paint layers. The painting is thus a composite material in which, when the ultimate load of the superposed layers is reached, the exposed areas of the textile undergo local stress concentrations that complicate the interpretation of the curve leading to overall failure. A tensile test procedure aimed at the elastic modulus of the entire stratigraphy, i.e., before reaching the failure of individual layers, has been privileged in the present study because it allows the behavior of the painting under normal conditions to be described. The elastic modulus of the whole stratigraphy can be used in predictive finite element simulations and to compare different materials. Such low strains and loads occupy only a small part of the plot when testing to the ultimate load and are easily blurred in the initial reorganization of the sample when the test is started. Tensions ranging between 1 and 3 N/cm are generally considered to be typical and not harmful for most paintings on canvas [3], [21], [22]. Under such test conditions, strains are typically less than 5%, with very little lateral dimensional change in the clamped specimen. In a symmetrical test system, the long compensating arms of the cruciform samples described in the literature become unnecessary, and only the material required for clamping is required in addition to the specimen to be tested.

### Samples dimensions

For research in conservation the possibility of testing small samples is crucial, because naturally aged materials (e.g., an old painting) are unique, variable according to local characteristics, and very scarce. Sample size is connected to both the dimensions of the machine and the forces involved. International standard procedures exist on uniaxial tests (ASTM, UNI, DIN) and are generally based on a common value of 1″ or 25 mm for the width of the sample. If a 25 mm square sample can be easily cut in new canvases, this is often unrealistic for naturally aged samples, also because the number of replicas implies statistical relevance. A sample width of 1 cm for naturally aged materials was chosen as a reasonable balance^4^ between their morphological heterogeneity and their inherent rarity.

### Testing loads, speeds and run limitations

Testing up to 30 N/cm (10 times more than the paintings' normal working conditions) provides a wide enough range of data, implying a very small force build-up on the machine’s structure^5^. Nevertheless, the structure was designed to withstand forces up to approx. 1000 N without undergoing relevant deformations. Testing speed influences the response of viscoelastic materials, as fast loading increases reaction force. Test speed must be adjusted to specific test conditions within a relatively wide range of velocities. In the case of a 5% strain for a 10 mm sample at the speed of 0.2 mm/min, the 0.5 mm run will be covered in 2.5 min. Assuming a theoretical sampling rate of 8 samples per second for force values, such a duration would detail the force path with 1200 readings, which is a more than adequate number. At the opposite end of the speed range are the peel tests, for which previous research [23] has proposed a reference speed of 100 mm/min. Elongation is to be measured with a precision of about 5 µm, in order to provide data for low-strain tests. Aiming at higher resolution would be impractical as it would require a very accurate environmental control for the machine's dimensional stability. The run of the clamps is limited by the length of the linear rails, and the choice was made to use 300 mm rails ensuring an overall run of approx. 400 mm in each direction.

### Testing procedures

The main tests that prove useful for conservation purposes^6^ are the following.

1. Low-stress tensile test. The test is designed to analyze the mechanical behavior of a painting facing stresses that are as similar as possible to those it would undergo in a typical conservation environment and conditions. The reference standard will be ISO/AWI 13118, once published. The sample is pulled at a constant speed until reaching a given target force. The test will finish when the force is reached or, as a safety feature, if a maximum distance of 250 mm is covered during the test. The target force and maximum distance are the values that can trigger the termination of the test procedure.
2. Yield and failure test. The test is designed to analyze the mechanical behavior of a painting under extreme conditions. The reference standard will also be ISO/AWI 13118. The sample is pulled at a constant speed while the force is recorded. The test is terminated when the measured force falls −70% from the recorded maximum value (due to fiber breakage of the specimen). As a safety feature, a maximum distance of 250 mm is allowed during the test. A target distance and target force are also variables that can trigger the termination of the test procedure.
3. Constant load test. The test is designed to measure creep and similar behaviors under a specified load^7^. The sample is pulled at a constant speed until reaching a given target tension. The machine keeps the tension constant by moving the clamps if needed. The elongation and force are recorded. This test can last a long time (hours) and might be performed in an environmental chamber so the sample response to changing environmental conditions can be analyzed.
4. Constant elongation test. The test is designed to measure changes in the tension produced within the sample over time after an initial load^8^. The sample is pulled at a constant speed until reaching a given target force. The machine does not move the clamps but records tension as it evolves. Changes of the environmental conditions, or the simulation of conservation treatments, can be associated with the test.
5. Uniaxial tensile tests. Tests designed to analyze the mechanical behavior of paintings material samples, as done with a uniaxial testing device. All the previously described tests can also be performed uniaxially, using only one pair of opposing actuators. Standards for uniaxial tensile tests for canvas are in ISO 13934.
6. Peel test. Peel tests are used for the characterization of the adhesive bond and are considered a reference for studies on lining techniques for canvas paintings. The test is a special case for uniaxial testing, intended to determine the bond strength of the adhesive used to bind together two textiles (in treatments such as lining). Two opposing clamps will tear apart the adherends at a set speed while the force is recorded. The test will end when the target distance is reached. The Peel test is described in the ASTM D 903 – 98 (Reapproved 2004), and a maximum travel distance of 350 mm^9^ is sufficient.

### Mechanical design

**Fig. 1:**
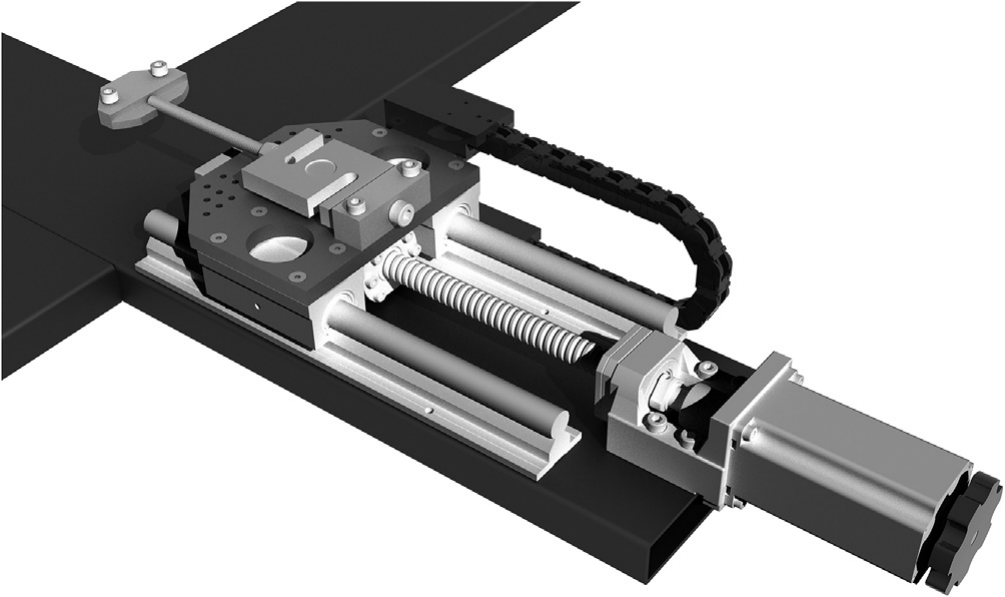
CAD drawing of one of the four actuators. From left to right: the sample clamp connected to the S-shaped load cell fixed to the carriage moving on the linear rails thanks to the ball nut (under the carriage) and ball screw turned by the stepper motor, with an additional knob for the manual motion.

The reference structure of the machine is a stiff metal cross to which four identical actuators are connected. Using a ball screw and ball nut for traction allows it to work in a condition that makes backlash less relevant than with other methods and allows to indirectly measure the elongation of the sample by counting the number of revolutions of the shaft. Stepper motors allow an accurate knowledge of position, provided that the forces involved do not exceed their torque, as this would make them lose steps. For the force measurement, load cells were seen as the most convenient sensor type because they are reliable and offer a robust mounting structure for the clamps. The mechanical design process was iterative: first, a proof of concept was implemented using components readily available in the workshop in Rome. An M12 threaded rod and a sliding door guide were used, all coupled to a stepper motor. That was the foundation of a one-axis actuator prototype that allowed us to start with a physical model for which we could write code and obtain test results. It should be noted that the entire process of designing and building the prototype and the final machine was done by working remotely in Spain and Italy. Most of the work was carried out during the COVID-19 lockdown in Europe. Sourcing the parts and obtaining semi-finished parts, laser cutting or machining from contractors was more challenging than usual. In the final design, each of the four arms of the cross houses an identical actuator consisting of a stepper motor^10^ and a recirculating ball screw^11^, which moves a carriage on two parallel linear guides^12^. Connected to each carriage is a load cell^13^ for force measurement and a clamp to hold each sample edge. The symmetrical design increases accuracy, as displacement is determined by two motors on each axis, and forces are read by four load cells^14^. Such a design also allows high flexibility of use. A CAD drawing of one of the four actuators of the biaxial tester is shown in Fig. 1. A ball screw and ball nut have been used to drive the motion instead of a threaded rod because of their greater smoothness, lower friction, and almost complete absence of play due to the preload of the ball bearings the ball nut rides on. These are definitely a more expensive choice but they were chosen because of their superior performance. Besides, the efficient elimination of the backlash means that sample elongation can be measured precisely through the angle of motion of the ball screw. Such a feature avoids the need for additional methods for measuring sample elongation. The pulling action of the machine is divided between pairs of actuators cooperating with simultaneous action on the same axis, so each produces only half the required speed and displacement.

### Mechanical construction

**Fig. 2:**
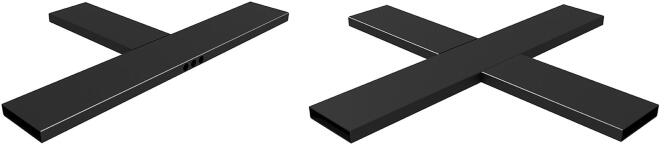
CAD drawing of the parts of the steel cross, made by welding two short elements to a long one, with holes on the side for the passage of the cables.

The cross was built by welding 150 × 30 mm, 3 mm thick standard steel profiles. In order to compensate heat-related tensions, welding^15^ was performed by gradually adding weld spots on opposite sides, a careful procedure that allowed for obtaining a perfectly planar structure. Before welding, holes were drilled on the two sides of the main element of the cross, under the connection with the short arms, in order to allow cables to pass through the structure (see Fig. 2).

**Fig. 3:**
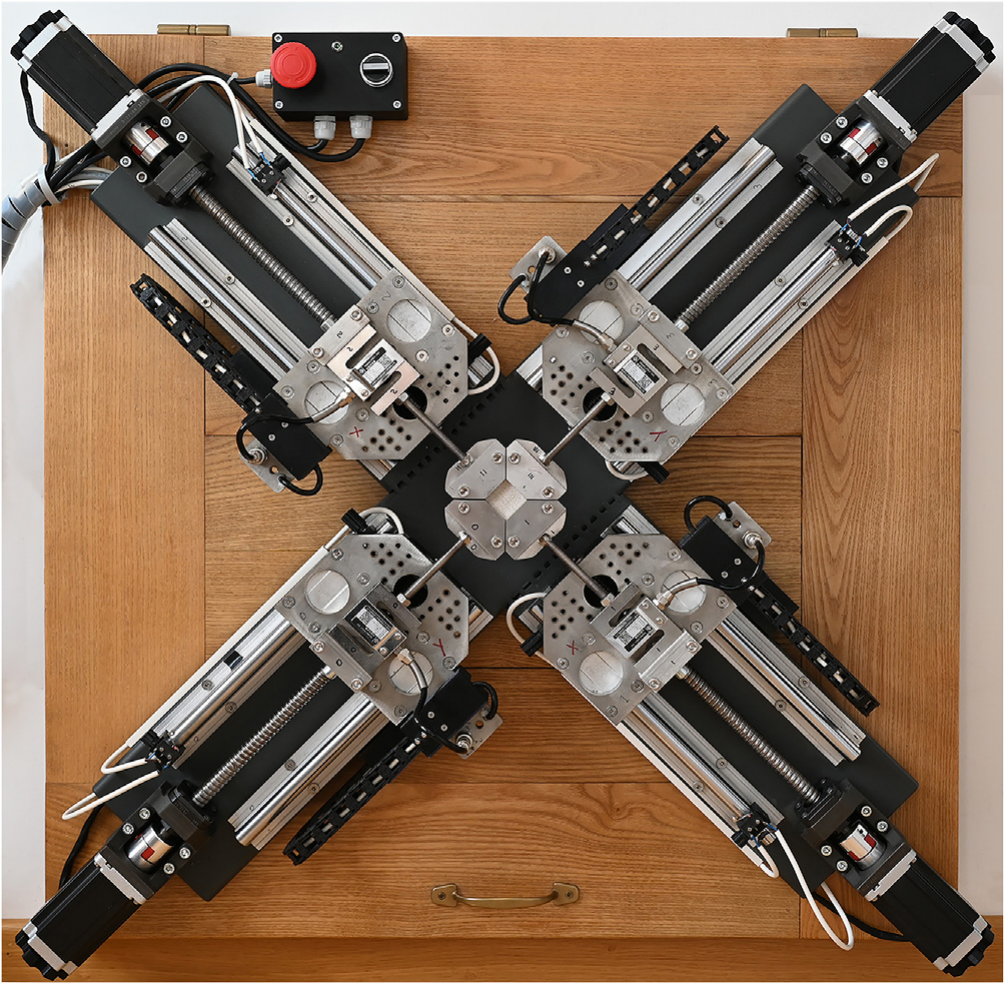
The biaxial tester assembled on a wooden table, with the control box for the security switches.

The overall dimensions of the cross, 91.9 cm, were determined by the length of the actuators, leaving a free 17.5 cm square area in the center for positioning and clamping the samples. All parts were mounted on the welded structure using stainless steel screws and bolts in threaded holes in the cruciform structure. After a complete cycle of testing the mechanics and the alignment of the moving parts, all elements were disassembled to powder coat the cruciform structure with a Polyester – TGIC Free varnish^16^ to prevent corrosion, and then the machine was finally reassembled (see Fig. 3). Steel parts for the main cross, carriage plates, and clamps were sourced directly from suppliers, as were industrial laser cutting and powder coating services. Welding, precision mechanical assemblies and 3D printing of parts were carried out in the workshop of Equilibrarte ltd^17^, in Rome. The linear guides, all parts needed for motion actuation, the motors and most of the electronics were purchased through online stores. The total weight is approximately 45 kg.

### Description of the motion actuators

Stepper motors can lose steps, thus making the measurement of the carriage position unreliable when the force opposing their motion exceeds their value of maximum admitted torque. The NEMA 23 4Nm stepper motors were chosen because they display a remarkably high torque^18^ for their compact size at an affordable price. Each motor was mounted on a cast steel support, which also holds the ball screw support bearing. The motor shaft is connected with a flexible aluminum coupler to the anti-backlash ball screw and ball nut, which is supported at the opposite end by a ball screw support floating bearing.

**Fig. 4:**
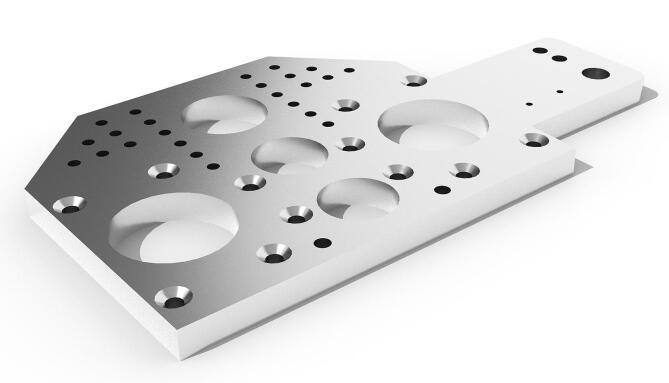
CAD drawing of the plate of the moving carriage, cut in 10 mm AISI 304 stainless-steel.

The ball nut is fixed with six bolts into an aluminum mount, providing a solid connection with the carriage plate on top of it. The carriage plates were laser cut from 10 mm AISI 304 stainless steel. Plates are connected to the 85 mm long open linear bearings running on the 16 mm diameter supported round rails in steel, as seen in Fig. 1, and holes were drawn for their connection to the linear bearings and the load cell supports. To reduce their mass by 20%, additional large holes were designed where they would not affect their rigidity (see Fig. 4). Protruding lateral support was designed to house the load cell amplifier board^19^ protected with a 3D printed box that also allows connecting the cable chain carrying the digitized load cell signal wires to the Arduino, as described in the next section.

### The load cells and clamping devices

**Fig. 5:**
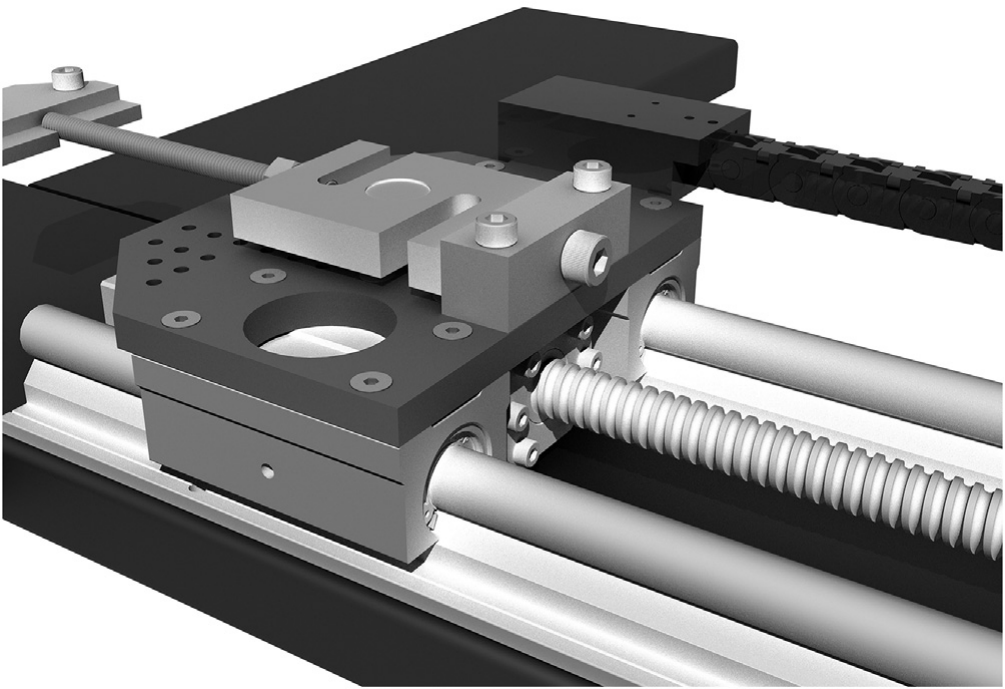
CAD drawing detail of the moving carriage showing the load cell on its support and the transverse bolt holding it.

The load cells are mounted on supports manufactured from a 20 × 20 mm bar of AISI 304 stainless steel (see Fig. 5). Two vertical holes connect them to the moving plate with M6 bolts, while a transverse hole connects the load cell with an M8 bolt. Load cell bolts are tightened with a dynamometric wrench at 5 Nm in order to reduce possible sources of different behavior.

Three series of load cells^20^ can be mounted on the machine with different load ranges. Load cells are in class C3, and manufacturer’s specifications are as follows. Sensitivity: 2.0 ± 0.05 mV/V; Linearity: ±0.03% FS; Repeatability: ±0.03% FS. The load cell signal is digitized and amplified with an HX711 board, which is placed on the side of the moving carriage in order to reduce the analog cable to a minimum length. Since multiple load cells can be used, a 4-pin screw connector is used in order to ensure stable contact and replacement ease. The digitized signal runs in a cable chain to protect the wires, then it is sent to the main control board through the machine structure, along with all the other cables, as will be described in the electronic design section.

**Fig. 6:**
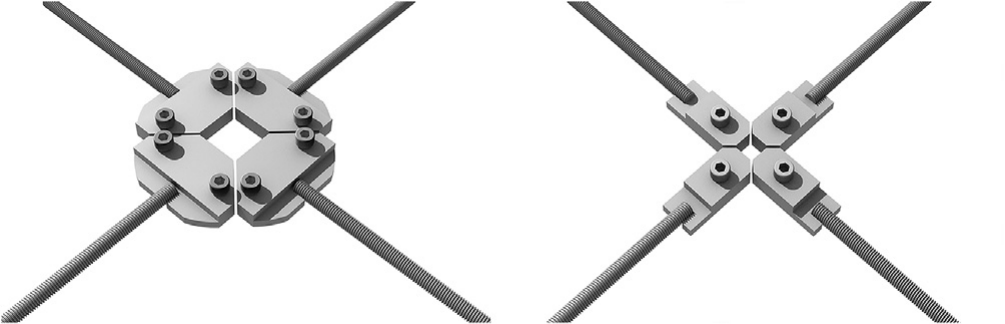
CAD drawing of the 25 mm and 10 mm sample clamps. The M8 rod is welded to the base plate, and the sample holder is fastened with M6 bolts.

**Fig. 7:**
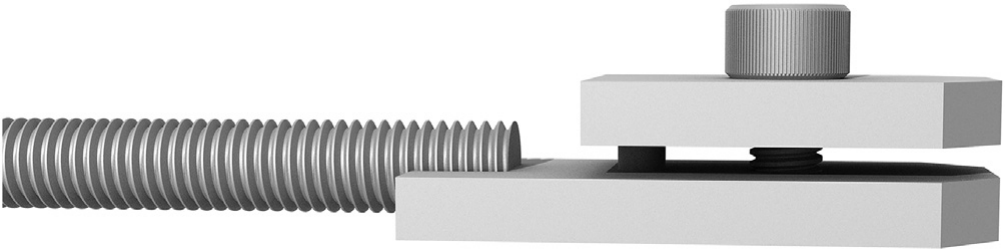
CAD drawing detail of the 10 mm sample clamp. The sample holder is guided with a 3 mm pin and fastened with a single M6 bolt.

The sample clamps (see Fig. 6, Fig. 7) are connected to the M8 ending of the load cell and are designed in order to keep the sample centered on each axis. Two AISI 304 plates are used, one welded to the M8 bar and the other serving as a sample holder, connected to the former with M6 bolts. The shape of the plates is designed in order to reduce the space between the clamps without reducing their rigidity. Therefore, 45 degrees cuts allow the edges to come close together, reducing the gage length to a minimum value very close to that of the sample area^21^.

**Fig. 8:**
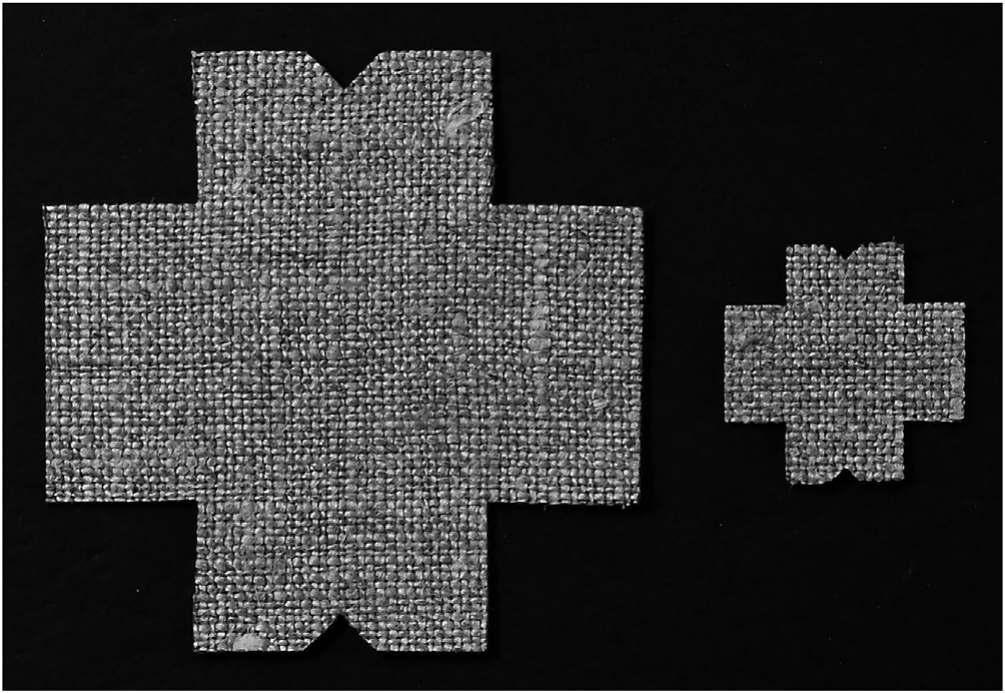
Laser-cut canvas samples, for the 25 mm and 10 mm clamps. The triangular indentation identifies the weft direction.

Samples are laser-cut to achieve a clean, linear perimeter and to avoid the mechanical stresses in the samples that may occur when cutting them with blades or scissors. As the directions of warp and weft are crucial information that may be lost once the sample is extracted from the material it was cut from, a triangular indentation in the perimeter of the clamping area allows identifying the weft (see Fig. 8).

### The end stops

**Fig. 9:**
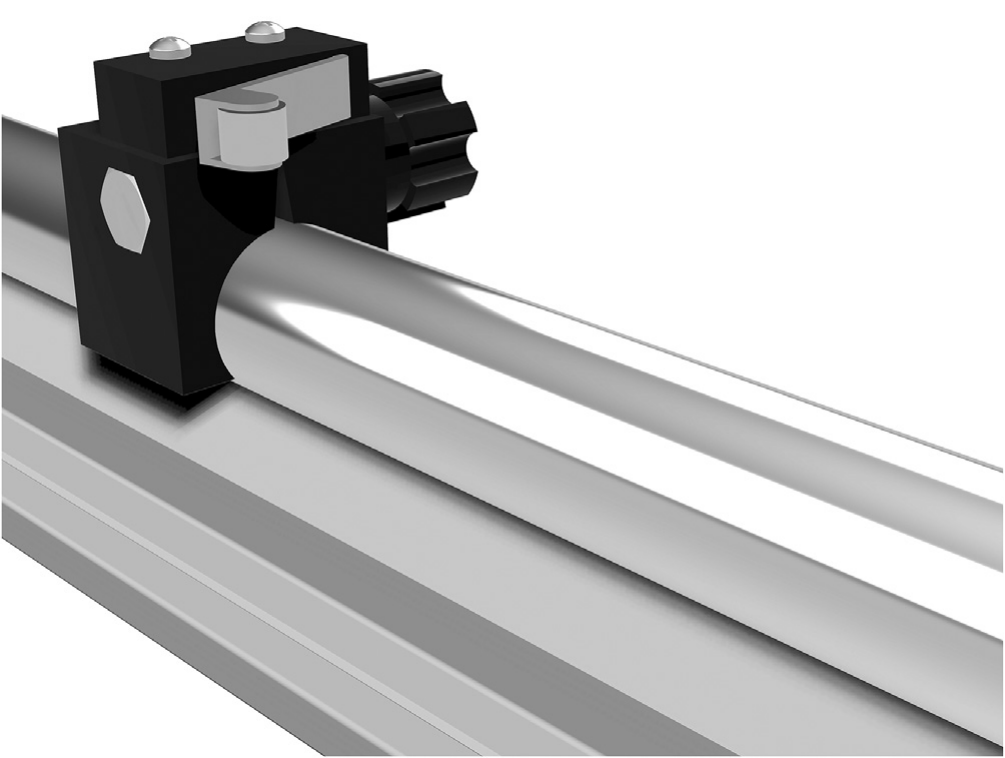
CAD drawing detail of a limit switch clamped on the linear rail.

To avoid collision of the carriages with the physical limits of the machine (i.e., motor mounts at one end of the linear rails and other carriages or clamps at the other end), limit switches are used to delimitate their run by sending an electrical impulse to the main control board. The limit switches are fixed on 3D-printed supports mounted on linear rails (see Fig. 9). As the supports can be easily unlocked and moved along the rail, such delimitation is also used for the homing process, a procedure allowing a temporary reference position for the carriages to be created. Such an additional tool proves extremely useful to replicate the starting point when a series of identical tests is to be repeated.

### Electronic design

**Fig. 10:**
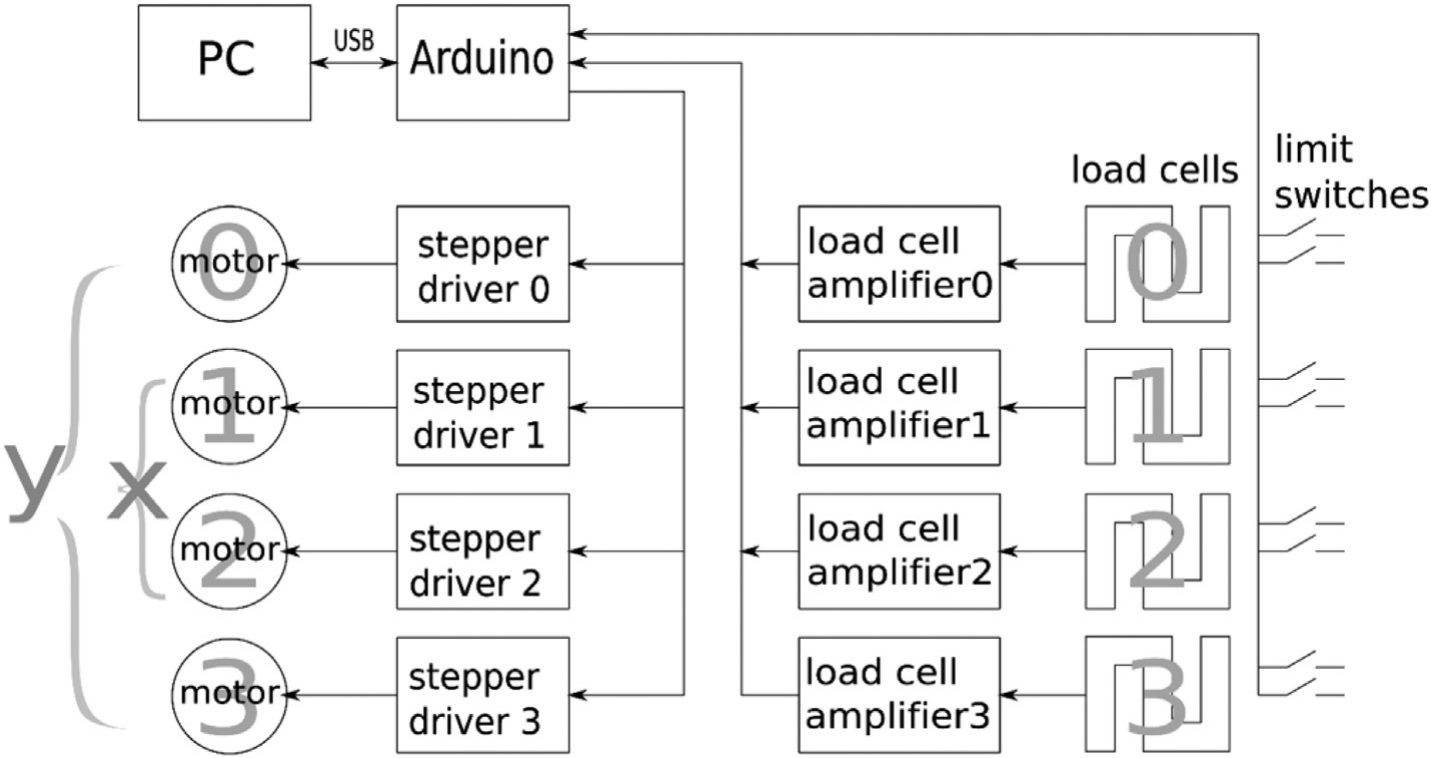
Schematic of the electronics.

Fig. 10 is a schematic of the electronics. A personal computer controls the whole testing process with the help of an Arduino microcontroller board that acts as a machine controller. The Arduino offloads some tasks from the PC, deals with time-critical signals and performs some data filtering too. It provides all the necessary inputs and outputs to interact with stepper motor drivers, the load cell amplifiers, which include a 24-bit analog to digital converter (HX711 chip), and the limit switches for the moving carriages. The four stepper motors used are numbered from 0 to 3, where motors 1 and 2 will pull from both sides along the X axis, while motors 3 and 0 will do the same along the Y axis. Each motor moves a carriage with an associated load cell and two end stops, each one at one end of the carriage travel. The personal computer is connected to the Arduino using a USB cable. Stepper motor drivers are powered by a 36 V power supply, while the other components are powered by a 5 V supply. Load cells and their amplifiers are powered with a separate 5 V power supply unit^22^, providing very low ripple/noise (2 mV RMS).

### Software development

Stepper motors allow an open-loop control of the motion of the carriages. In a nutshell, a stepper motor moves in small-angle increments called steps. These steps can happen clockwise or counterclockwise depending on a digital signal called direction. The controller sets the direction input and supplies the number and frequency of step pulses so the motion will happen in the desired direction at the set speed. For a constant speed, the step pulse frequency will be constant too. Once the choice of using stepper motors and their drivers is made, a machine controller is needed to provide all the signals to move each motor while it can read all the sensors to precisely control the biaxial tester operation. Different approaches are possible, like creating a stand-alone controller that would include the required user interface to operate the machine or a split approach where a personal computer would run the user interface that will interact with a simpler machine controller. It was this latter approach the one that was selected as it seemed easier to replicate by others. Software development involves the creation of two different elements. On the one hand, the machine controller and firmware, built and developed around a microcontroller that will interface directly with motor drivers, load cells, and limit switches. On the other side, the biaxial tester will be operated from a personal computer with a second piece of software that will communicate with the machine controller and display data as real-time graphs on the display within the graphical user interface.

### Machine controller firmware

As soon as we had the first mechanical one-axis prototype, the next development step was to create a machine controller that could handle all the electronics and create the proper signals for it to work as desired. Generating the stepper motor signals was one of the tasks, but reading the force of the different load cells would be another. A third task of the controller is to accept commands from a computer and to send all the measurements performed to it, so the data of an experiment can be stored, graphed, and interpreted. Given these basic requirements, using an Arduino board was the first choice for such a controller and an 8-bit Arduino UNO proved to be sufficient for the task.

The regular operation of our biaxial test machine is to perform a battery of tests on several samples. Each test consists of an initial setup where the sample is clamped to the machine and centered in the cross. This requires some motion of the carriages that can be done by hand if stepper motors are disabled, or by using a collection of pushbuttons on a Graphical User Interface (GUI) in the PC program that controls the biaxial tester. Once the sample is appropriately positioned, an initial tension is applied to it to produce a standard starting condition (“pre-tensioning”); the test will begin whenever the user commands it. While the test takes place, the computer will receive the measurements performed and transmitted by the controller. Depending on the test, that information is just stored in a file, or it can also be displayed as a graph on the computer screen. Real-time graphs and video recordings of the sample can give the researchers a clear view of what is happening.

The controller software will continuously be connected and operated via a computer. The controller software will not have many initiatives but, on the contrary, will always be commanded from the computer it is connected to. The typical connection from the computer to the controller is a USB connection that emulates a serial port connection in the case of the Arduino boards. Therefore, the controller software will be a basic command interpreter that waits for a new command to be received over the serial port. Commands are all a single line of text ended by a carriage return (see Appendix 1). Once a command is received, it is parsed, and the command is executed. Depending on the command, the response is immediate or takes a long time. Depending on the command, there might be some data transmitted to the computer by the controller while a command is being performed (i.e., a peeling test will move the motors while it transmits back the measured forces on the load cells). Every time a new serial connection is opened from the PC, the Arduino board is reset, and the controller program starts with the default values for variables like home speed, test speed, elongation, maximum force, etc. These values can be changed from the PC software with the suitable command from the list above. Please note that in the current implementation, the firmware default values are hardcoded, and any changes will vanish once the controller is reset. It may be a surprise that there are no separate commands for each type of test the machine can perform. For each type of test, a sequence of the existing commands is all that is needed, and the concatenation of several commands to make a test will be done by the PC software.

### Pre-tensioning algorithm

Biaxial tests describe the mechanical behavior of a sample and the interaction between warp and weft. It is therefore crucial that the initial conditions are as close as possible to a desired balance of forces before starting a test. The ideal condition is that tension is equal in the two axes, which is difficult to achieve because the sample reacts in both directions when an action is performed on one. An algorithm has been developed so the controller firmware can perform this initial adjustment much quicker and more accurately than a human. In cases where the test is uniaxial, the algorithm applies only to the actuators of the chosen axis.

The algorithm can be described as follows:

1 For each actuator, probed in a clockwise direction, a load cell measurement will be taken. Depending on the measured force, an additional pull (that is, moving the carriage one impulse outward) will happen if that carriage force sensor does not experience a pulling force higher than the pre-tension value set for that axis. However, if that force were exceeded by more than 10%, the carriage would move one step inward to compensate for that.
2 The process in 1 is repeated until either the desired pre-tensioning time has been exhausted or both axes have reached the desired pre-tension.

### PC software development

**Fig. 11:**
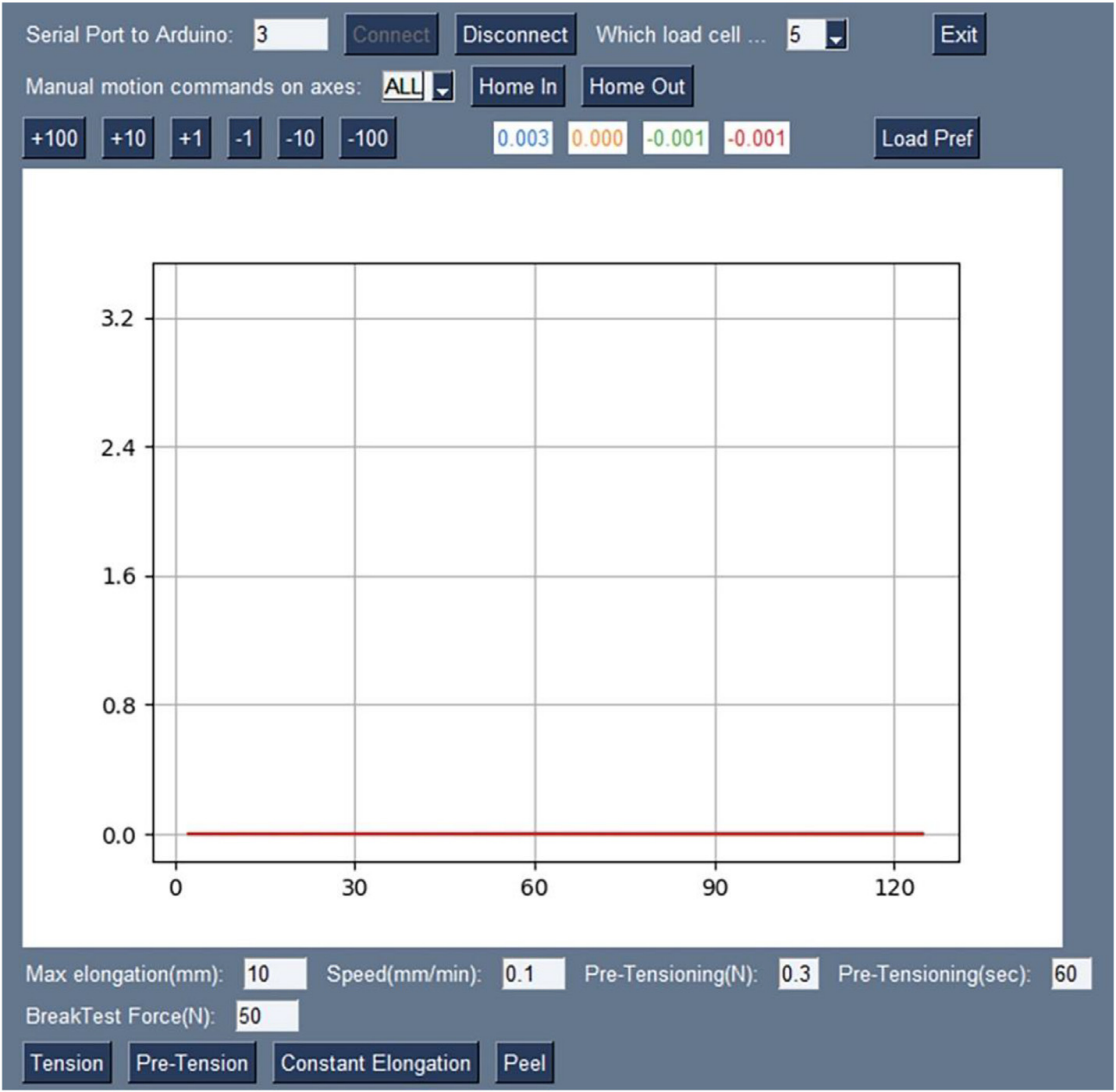
Screenshot of the user interface.

One of the first requirements for PC software was to make it multiplatform so that it could run on different operating systems. While it was not required, it would be nice if the program could offer real-time graphics of a test in progress. Various alternatives were considered, but development eventually focused on using Python3 and the PySimpleGUI library that, combined with Matplotlib, offered a simple way to deliver a GUI with excellent graphics for Windows, Linux, or OSX users, see Fig. 11. The general instructions are on the top line, the tests description and launching commands are on the bottom; in the middle of the screen is the plot of the readings of the four load cells, which are also shown as numbers in the four white spaces with colored text, above the graph.

The task of this program is to allow the user to prepare a test, load the sample on the biaxial tester, and perform a test. While the test is running, the machine controller will be streaming data to the PC software that will be used for the real-time graph shown in the program's GUI and later stored in a file for future uses. The program operator will perform a collection of tests on many samples. In many cases, the test methodology will call for the test to be repeated on a given number of samples, so we can be certain the observed behavior is highly repeatable and accurately represents the response of that material to the test within the desired margin of confidence. The biaxial tester may perform single-axis tests as well as biaxial ones. The user can perform manual moves of the carriages either individually, two carriages along the same axis, or four carriages at once for any configuration required for a test.

Let us see, as an example, how a low-strain biaxial tensile test (including pretensioning) is performed:

1 The user will start the PC software by clicking the program icon.
2 The proper serial port name will be selected on the program GUI and communication with the machine controller will start when the user presses the “Connect” button.
3 As soon as the connection is made, the values of the load cells readings will be zeroed (by means of the "T" command sent to the Arduino board) the real-time graph on the GUI will start to be updated as samples are collected from the four load cells (by means of the “?” command).
4 Using the home button (“O” command) and perhaps the manual motion buttons (“X” command), the user will locate the clamps to the initial position, and then a new sample will be placed in the clamps that will be properly tightened.
5 The user will pre-tension the sample using a specifically developed procedure assuring that the sample will be subject to a defined tension value, by operating independently on each axis. Pressing the “Pre-Tension” button will trigger the setting of the pre-tension forces (commands “K” and “J”), and then the pre-tension algorithm is started (command “W”) for a specified amount of time. The test will finish when the desired tension is reached on all axes or if the maximum time is reached. The user can see on the real-time graph whether the case was the former or the latter and act accordingly.
6 The user will place the desired values for the test speed (command “V”) and maximum elongation (command “L”), and next, will press the “Tension” button. This will set the maximum stopping force (command “C”) and the motors involved (command “M”) that will be the four of them for a biaxial test. Next the speed of the motors is set (command “V”) and the elongation is reset (command “R”). Finally, the test motion is started (command “X”) until either the maximum force or the maximum elongation is reached. The graph in the GUI will show the real-time evolution of the test.
7 Once the test is over, all received data is stored in a file in a given folder for future use.

### File format

Data obtained from any test is stored in text format as comma-separated values (CSV file). Using CSV format makes it easy to process data later using other programs such as Microsoft Excel or Gnuplot.

Each line on the CSV file contains six different values:

1 Timestamp in milliseconds: this is the number of milliseconds since the PC was connected to the Arduino, and it changes for each line, increasing monotonically.
2 Elongation in millimeters: this is the total distance the sample has been pulled at this moment. For very low-speed tests, it is possible to get several lines with the same elongation, meaning several measurements are taken for the same elongation.
3 Load cell 0 force in Newtons: this number is positive for a pulling force (that will be the case for most tests).
4 Load cell 1 force in Newtons.
5 Load cell 2 force in Newtons.
6 Load cell 3 force in Newtons.

For the sake of simplicity, the file format does not change whether a uniaxial or biaxial test is performed. For any uniaxial test, only two load cells will show useful values.

In the current iteration of the tester, the elongation value is an inferred one. Elongation is based on the theoretical distance between the two opposing clamps as a result of the number of steps the stepper motors have moved from the beginning of the test. Validation tests show us that absolute error is of the order of a few microns.

3. Design files summary.

**Table:** 

Descriptor	File name in repository https://zenodo.org/record/7541996#.Y8Wd1uLMIqs
Firmware	prototype-4motors-newPreTv2_ver0.13.ino
	biaxialPref.ini
	version_tracker.h
Pc software	speed_increased2.py
Electrical connections in the machine	Schematic_Biaxial_Tester.pdf
Cad of the entire machine	biaxial_tester.stp
Steel cross	steel_cross_holes.stp
Box for the safety switches	switch_box.stp
Sample temporary supports	sample_rest.stp
Manual motion knob	motor_manual_motion_knob.stp
Cable chain support, top	cable_chain_support_top.stp
Cable chain support, bottom	cable_chain_support_bottom.stp
Carriage plate	carriage_plate.stp
Limit switch supports internal	lim_switch_int_support.stp
Limit switch supports external	lim_switch_int_support.stp
Limit switch knobs - internal	lim_switch_int_knobs.stp
Limit switch knobs - external	lim_switch_ext_knobs.stp
Sample clamps – 10 mm	10mm_clamps.stp
Sample clamps – 25 mm	25mm_clamps.stp
Descriptor	web link for download
Ballnut Floating Bearing datasheet	https://www.cnc4you.co.uk/Ballscrew-Support-BF15-TMT-(SYK)?search = ballscrew%20
Ballunt datasheet	https://www.cnc4you.co.uk/Ballscrew-with-Fitted-Anti-Backlash-Ballnut-RM1605-C7-16 mm?search = ballnut
Ballscrew support datasheet	https://www.cnc4you.co.uk/BK15-C3-SYK?search = ballscrew%20
CW5045 Driver Wiring Diagram datasheet	https://www.cnc4you.co.uk/Stepper-Motor-Driver-4.5A,-50 V-CNC-Microstepping-CW5045?search = CW5045%20
CW5045 Driver datasheet	https://www.cnc4you.co.uk/Stepper-Motor-Driver-4.5A,-50 V-CNC-Microstepping-CW5045?search = CW5045%20
Ballscrew support nut datasheet	https://www.cnc4you.co.uk/Ballscrew-Mount-DSG16H-ID-28 mm?search = dsg%20
Power Supply Unit datasheet	https://www.cnc4you.co.uk/400 W-PSU-36Volt-11Amp?search = power%20supply
Complete linear rails datasheet	https://www.cnc4you.co.uk/Linear-Rail/Supported-Round-Rail-Kits/SBR16LUU-Rail-Kits
Stepper motor datasheet	https://www.cnc4you.co.uk/Stepper-Motor/Nema23-4Nm/Stepper-Motor-4Nm-60BYGH401-03-Nema23

4. Bill of materials summary.

**Table:** 

Component	Units	Unit cost	subtotal	Source of material	Model/material type
Cruciform structure	1		€ 40.00	Hardware shop	Carbon steel
Welding	1		€ 200.00	In studio work facility	TIG welding
Powder coating	1		€ 40.00	Industrial powder coating service	Polyester
Stepper motor, NEMA 23, 4 N/m	4	€ 39.19	€ 156.81	https://www.cnc4you.co.uk/	60BYGH-401–03
Motor Bracket SYK	4	€ 37.49	€ 149.98	https://www.cnc4you.co.uk/	MBA12-C-SYK
Stepper motor driver	4	€ 44.04	€ 176.15	https://www.cnc4you.co.uk/	CW5045
400 W 36 V 11A power supply	1	€ 45.57	€ 45.57	https://www.cnc4you.co.uk/	400W36V/11A
5 W 5 V 1A power supply	1	€ 87.00	€ 87.00	https://www.oep.co.uk/	PS2125
Ball screw with anti-backlash	4	€ 76.37	€ 305.47	https://www.cnc4you.co.uk/	RM1605-C7
Ball screw support # 1	4	€ 38.26	€ 153.03	https://www.cnc4you.co.uk/	FK12_TMT_(SYK)
Ball screw Support # 2	4	€ 23.78	€ 95.12	https://www.cnc4you.co.uk/	BF12_TMT_(SYK)
Ball screw Mount	4	€ 11.39	€ 45.55	https://www.cnc4you.co.uk/	DSG16H
Supported Rail Kit	4	€ 84.04	€ 336.16	https://www.cnc4you.co.uk/	SBR16LUU_Rail_Kit
XD Coupling	4	€ 13.05	€ 52.21	https://www.cnc4you.co.uk/	XD_30mm_X_35mm
carriage plates	4	€ 9.50	€ 38.00	https://www.inoxcadei.it/	AISI 304 stainless steel
25 mm sample clamp	4	€ 7.00	€ 28.00	https://orrilamiere.it/	AISI 304 stainless steel
10 mm sample clamp	4	€ 5.00	€ 20.00	https://orrilamiere.it/	AISI 304 stainless steel
Cable chain	4	€ 9.00	€ 36.00	https://www.igus.it/	E.2.10.10.038.0
Terminal Block Shield Board	1	€ 21.99	€ 21.99	https://www.amazon.it/	Wingoneer T0339
Load cell amplifier	4	€ 1.60	€ 6.40	https://www.amazon.it/	HX711
Arduino	4	€ 22.00	€ 11.00	https://store.arduino.cc/	Arduino Uno
5 kg load cell	4	€ 23.52	€ 94.08	https://www.aliexpress.com/	C3 class load cell
50 kg load cell	4	€ 23.52	€ 94.08	https://www.aliexpress.com/	C3 class load cell
100 kg load cell	4	€ 29.41	€ 117.64	https://www.aliexpress.com/	C3 class load cell
4-pin connector	9	€ 2.00	€ 18.00	https://www.amazon.it/	
6-pin connector	4	€ 2.75	€ 11.00	https://www.amazon.it/	
Limit switch	4	€ 0.50	€ 2.00	https://www.amazon.it/	GTIWUNG T331
Emergency stop button	1	€ 12.50	€ 12.50	https://www.amazon.it/	Durlclth62egafyswn
Motor disable switch	1	€ 12.50	€ 12.50	https://www.amazon.it/	Tyenazagm375pyku6
Cables		€ 30.00	€ 30.00	Hardware shop	
Limit switch supports - internal	4	€ 0.75	€ 3.00	designed and built in studio	PLA 3D printed part
Limit switch supports - external	4	€ 0.75	€ 3.00	designed and built in studio	PLA 3D printed part
Limit switch knobs - internal	4	€ 0.75	€ 3.00	designed and built in studio	PLA 3D printed part
Limit switch knobs - external	4	€ 0.75	€ 3.00	designed and built in studio	PLA 3D printed part
Manual motion knob	4	€ 0.75	€ 3.00	designed and built in studio	PLA 3D printed part
Cable chain support, top	4	€ 2.50	€ 10.00	designed and built in studio	PLA 3D printed part
Cable chain support, bottom	4	€ 2.75	€ 11.00	designed and built in studio	PLA 3D printed part
Box for the electronics	1	€ 30.00	€ 30.00	designed and built in studio	Laser cut plywood
Total cost of materials and supplies			€ 2502.20		

## Build instructions

### Construction of the steel cross

The steel cross design is the result of a negotiation between weight and rigidity which proved easy to source and build and efficient for the model and the uses described. Before welding, it is necessary to perforate the hidden faces of the central element so as to allow the passage of all the cables. It is important to clean the inside of the hole with a file so as to remove any potentially cutting edge. A set of 3–20 mm- holes proved sufficient, but up to 6 holes could be drilled. The distortion-free welding process is not within reach of a beginner, but a professional could accomplish it for an affordable cost, as indicated in the Bill of Materials. Alternative solutions can be pursued for the main structure, such as using lighter metals or even carbon-epoxy composites, keeping as the only constraint the width of the face on which the linear guides rest, whose overall footprint is 150 mm.

### Perforation of the structure for fixing the actuators

The walls of the steel tubes with which the structure is constructed are 3 mm thick also to enable the holes to be threaded, for easy attachment of the superposed elements. The position of the holes must be transferred with great precision, so the use of the supplied CAD drawing is recommended. The transfer can be done through a paper print or a cardboard model made with a laser cutting machine. Linear guides require M5 screws, while the cast iron motor mount and the ball screw support require M6. It is recommended to use the CAD model to make small guide holes (2.5 or 3 mm) and then enlarge to the required diameter for the necessary threading (4.2 and 5 mm, respectively).

### Test the assembly of the actuators and powder coat the structure

Before having the structure powder coated, it is recommended to test the assembly and motion of the four actuators and make any connection holes with external secondary structures, such as a tabletop. As the final preparation for powder coating, it is recommended that all roughness and surface defects on the steel be removed and the steel is thoroughly cleaned with degreasing substances.

### Installation of the cables and of the actuators.

The load cell cables will not be subjected to intense bending stresses, not only because they run in the cable chains but also because the movements of the machine are much slower than in industrial automation. Cable quality does not need to meet very high standards but needs to ensure a reliable connection. The cables need to be grouped within a spiral cable wrap. The carriage plates are designed with holes matching those on moving parts of the linear rails, plus two M6 placed to hold the load cell support, which shall be corrected according to the load cell model chosen for any future construction. The design is laser cut, and will require some extra work for manual tapping and countersinking a few of the holes.

### Construction of the sample clamps

Two series of sample clamp designs are provided for the 25 × 25 mm and the 10 × 10 mm samples. Both are made from laser cut 5 mm thick AISI 304 stainless steel, with a M8 threaded bar welded in a dedicated housing so as the axis of the sample plane corresponds to that of the load cell (which also has an M8 connection). Two M6 threaded holes are used to secure the top plate and sample.

### Construction of the box for the electronics

**Fig. 12:**
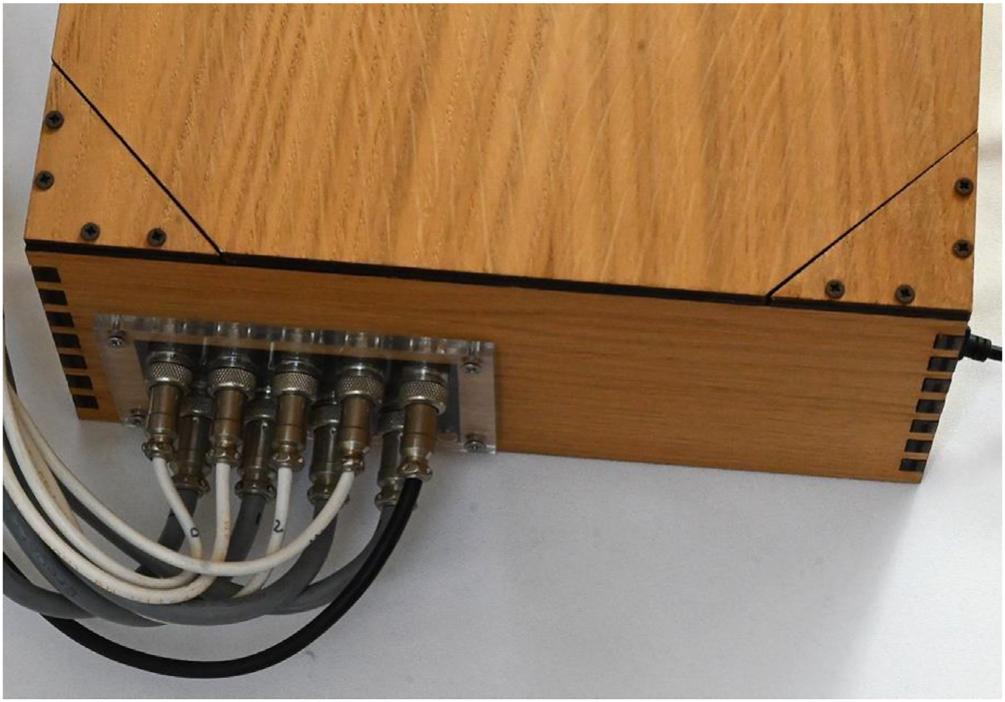
The pin connectors on the box for the electronics. On the right the USB cable connection to the laptop.

To hold the electronic components (stepper drivers, Arduino board, and power supply units) a dedicated box was designed, in veneered plywood with a thick aluminum bottom plate to help dissipate heat. On one side a grid is cut to favor air exchange from the PSU, on the other a hole is cut to house a Plexiglas plate designed to hold the pin connectors. The construction requires the availability of a suitable laser cutter for plywood and Plexiglas and simple hardware tools for the bottom plate and the assembly. But such a feature is not necessary as the electronics can be housed in any container or even simply fixed on a support or rail. The cables reach the box through pin connectors on a Plexiglas plate in order to make disassembly and transportation easier (see Fig. 12).

### The box for the switches

The red emergency button needs to be placed in an easily accessible location in the work space. It was decided to add an – optional – switch in the same spot to provide power to the stepper motor drivers, to simplify manual motions of the carriages with unpowered steppers, if any were necessary. A box to hold the two switches and for a green LED light was made in 3D printing and is visible in Fig. 3.

## Operating instructions

### Clamping the sample

**Fig. 13:**
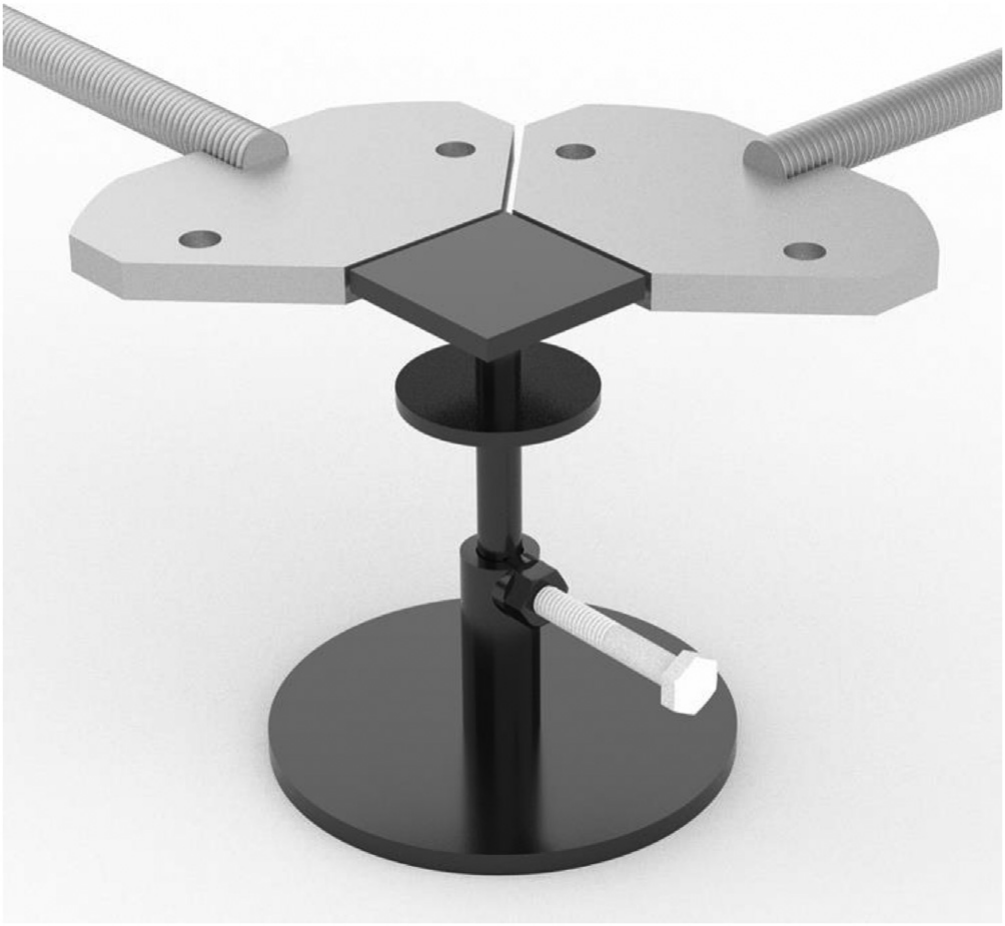
The adjustable temporary support for the samples to be used during clamping.

When clamping a sample, two aspects are very important: keeping track of the orientation of the textile and avoiding any kind of distortion, which would end up affecting the test result. Regarding the first aspect, it was decided as a convention to always place the warp between actuators No. 1 and 3. In order to avoid any distortion of very light and flexible fabrics, adjustable supports were 3D printed to set up a continuous support plane connecting the lower surfaces of the clamps, as in Fig. 13, where for clarity only the sample holding part of two of the four clamps is shown. This allows the specimen to be held in position, even with light finger pressure, while tightening the clamps. To prevent slippage of the fabric inside the clamps, which have a flat surface, it was found sufficient to make that surface somewhat rough by machining or to glue a layer of sandpaper.

### Test procedures tips

**Fig. 14:**
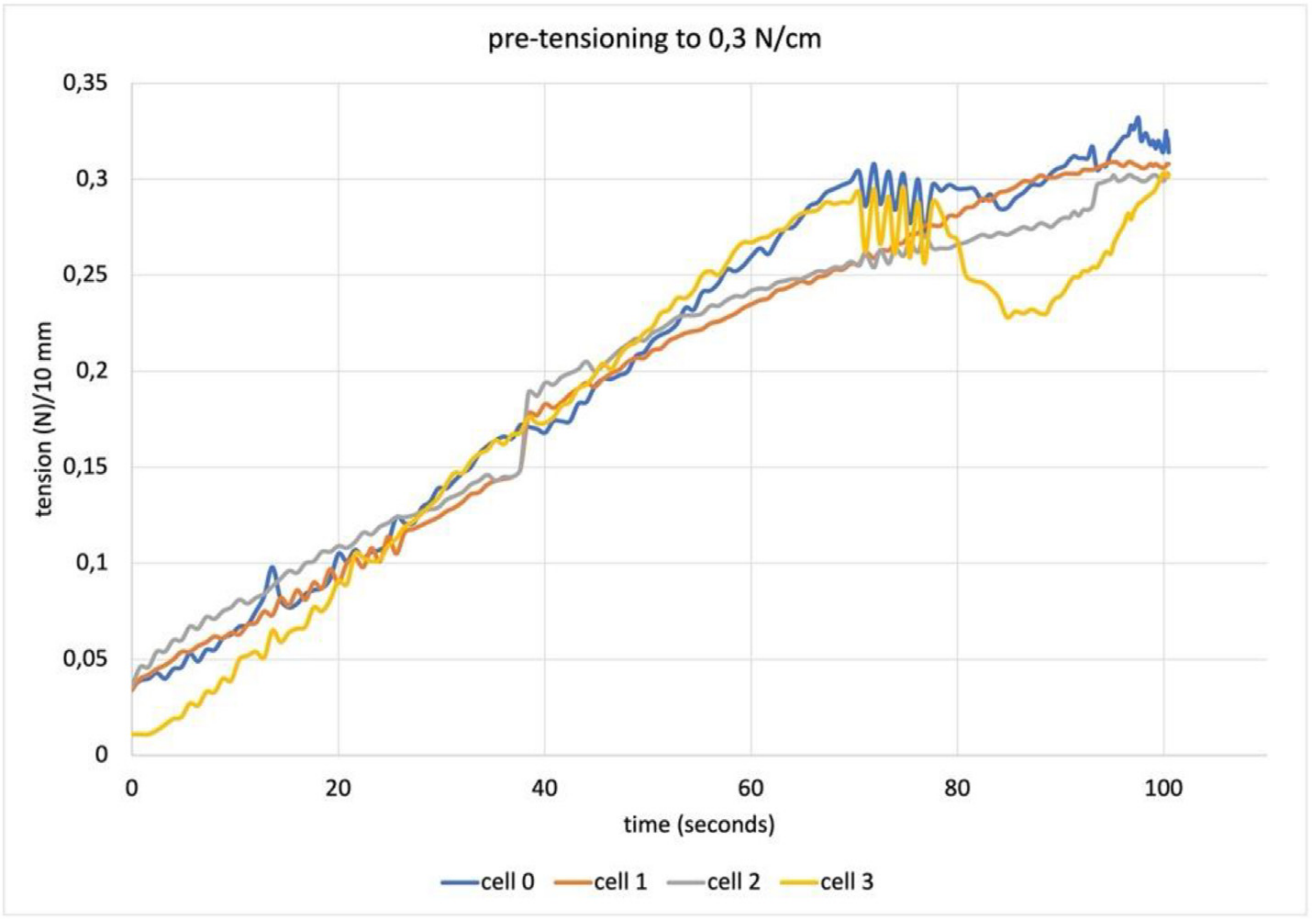
A typical plot for a pre-tensioning procedure with the target force of 0,3N/cm.

**Fig. 15:**
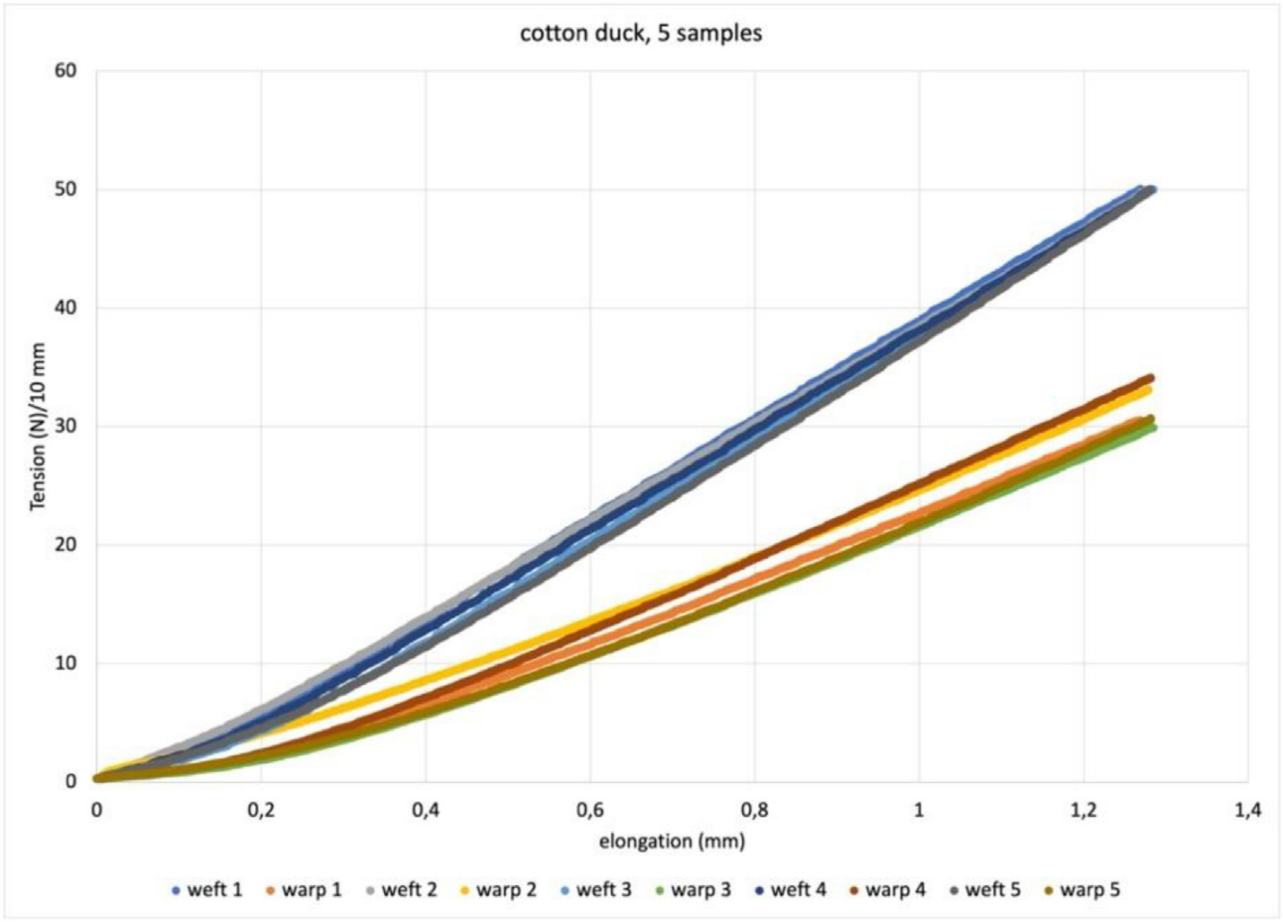
Plot for biaxial tensile test of 5 samples measuring 10 × 10 mm of the same cotton duck, starting from 0,3N/cm as obtained in Fig. 14, with the target force of 50 N.

The previous description of the software is already granting enough information about the different test procedures. We shall highlight here a few tips for the best use of software which is fully operational but can still be improved. After clamping the sample, the pretensioning procedure can be launched: a relatively long pretensioning time is recommended (at least 200 s), as the procedure will stop as soon as the result is obtained and it can be difficult to make a reliable prevision of the time it will require^23^. The chosen test procedure can be launched directly upon completion of the pretensioning (which produces a “pre-tensioning.csv” file keeping track of the entire operation). When the “homing” procedure is needed (either “home in” or “home out”) to reach a predefined position to repeat a test, restarting the software becomes necessary. It only takes seconds to reboot, but doing so offers the advantage of safely starting the new test. A video showing the screen interface during the pretensioning to 0,3N/cm (as in Fig. 14) and of the and biaxial tension of the sample (as in Fig. 15) is available at: https://youtu.be/p3qZJt3natk. Another video, showing the same procedures on the machine side, is available at: https://youtu.be/sCjQdkxGuNs.

## Validation and characterization

### Displacement of the carriages

The expected accuracy of carriage positioning is related to the angular rotation of the motor shaft due to a single pulse emitted by the driver and the pitch of the ball screw drive system. Each pulse at the chosen microstepping rate (16 microsteps) is producing a rotation of 1.8/16 degrees, that is 0.1125 degrees. The pitch of the ball screw is 5 mm for a complete turn; therefore, the expected motion for 0.1125 degrees is 0,00156 mm. We decided to use a digital dial indicator^24^ with 0.001 mm resolution to measure the accuracy and repeatability of the motion of the carriages under the control of the software. Such test would also verify the statements of the manufacturer of the parts. A 3D printed part was designed to keep the dial indicator fixed on the linear rail while the carriage moves towards and away from it. The motion was performed under the control of the user interface.

Two sets of commands were tested: the first was a 5-mm forward and 5-mm backward movement (each corresponding to 5760 microstepping commands); the second was a 10-mm movement obtained by repetition of two single 5-mm commands (to accumulate any errors in the procedure), followed by the same backward path. Both groups of movements were recorded in three series of 6 repetitions in each direction. Only the outward movements were analyzed, as these are involved in the actual testing motion during the machine’s use. Therefore 18 outward measurements for each carriage for each group were used, and a statistical treatment of motion data (a total of 144 recordings) yielded an average error with respect to the required displacement of 0.004 mm, with a standard deviation of 0.003 mm.

The homing process, which uses end stops as an additional tool for positioning the carriages in the same location, was tested for repeatability with a series of 12 consecutive homing movements for each limit switch. The typical average value of the 12 measures provides a positioning error of 0.005 mm, with a standard deviation of 0.001 mm.

### Load cells data

Load cell calibration and subsequent readings were done with a set of F1 precision weights: 100 g; 500 g; 1000 g and 5000 g^25^. The load cell readings were performed through the machine's electronics: the HX711 digitizing board, cables, connectors, and finally, the Arduino board and the PC software interface. After an initial calibration with the 1000 g mass, the load cells provided readings that correspond to the C3 class manufacturer’s specifications, thus indicating that the electronics does not introduce significant error (see Table 1).

**Table 1:** Measurements with the load cells.

Test mass (g)	Reading on screen (N)	Reading (g)	Difference (g)	Error (%)
100	0.973	99.218	−0.782	0.782
500	4.494	499.080	−0.920	0.184
1000	9.797	999.016	−0.984	0.098
5000	49.045	5001.198	1.198	0.02

## Results

### Pre-tensioning

The pre-tensioning procedure is based on continuous cycling attempts to bring the load cell reading on each actuator to a value that is close to the average of the four, and the average to the target value. The procedure is influenced by the stiffness of the material since the amount of force transmitted from one motor to the load cells on the others depends on the elastic modulus of the sample. An oscillatory behavior is intrinsic to the procedure itself and is broader when the sample modulus is higher. When the target value is reached as the average of the four, or the target pre-tensioning time has elapsed, the procedure is automatically ended. The sample plotted in Fig. 14 is an industrial cotton duck canvas: 0,72 mm thick, 350 g/m2.

Achieving an automated and relatively accurate pre-tensioning procedure is considered a major advance in materials testing for the specific field and a so-far unique feature of this machine. Manual pre-tensioning is easy for uniaxial samples, but experience says it would never achieve comparable levels of accuracy for biaxial samples. A finely adjustable procedure is crucial when dealing with low stress and strain values since the quality of any test performed subsequently is affected by the initial tensile conditions of the specimen. In our preliminary work we found significant differences in biaxial tensile test results using identical specimens with different pretensioning conditions.

### Biaxial and uniaxial tensile tests

**Fig. 16:**
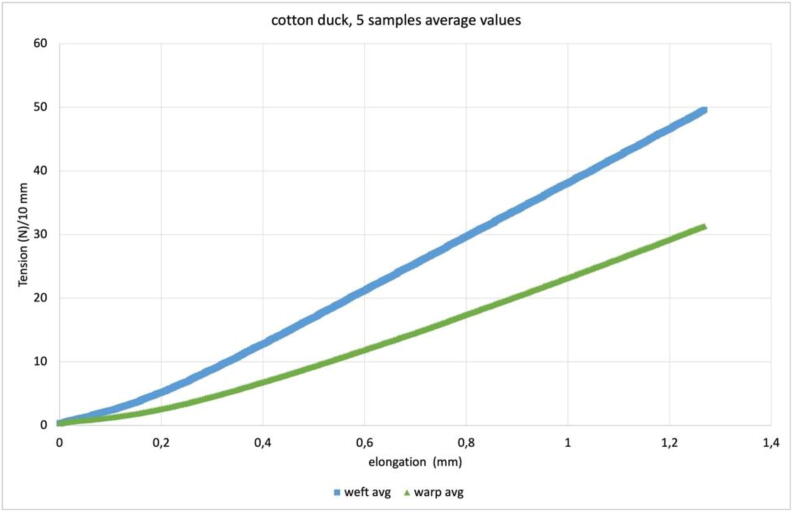
Plot of the average values of the 5 tests shown in Fig. 15.

The biaxial and uniaxial tensile tests are based on readings from the two load cells working at opposite ends of each axis. The availability of the four force values makes it possible to investigate any internal misalignment of the sample’s behavior, but the basic way to use them is to average the values of the load cells at opposite ends of each axis. In Fig. 15 we see the plot the average of the opposite load cells (in warp and weft) for 5 identical samples (10 × 10 mm) of the same cotton duck for which pretensioning is shown in Fig. 14. The pretensioning procedure is mostly depending on the manual clamping procedure it aims to even out; biaxial test plots are instead connected with the sample response to the symmetric action of the tensile tester. One of the sample directions will require a greater force for the same elongation, and some transfer of stiffness will occur, which will increase the tensile strength of the more yielding direction. Nevertheless, the more yielding direction can prove less constant in its characteristics, as is the case of the warp in Fig. 15. The plot in Fig. 16 is the value obtained by averaging the results of the 5 samples shown in Fig. 15.

### Peel tests

**Fig. 17:**
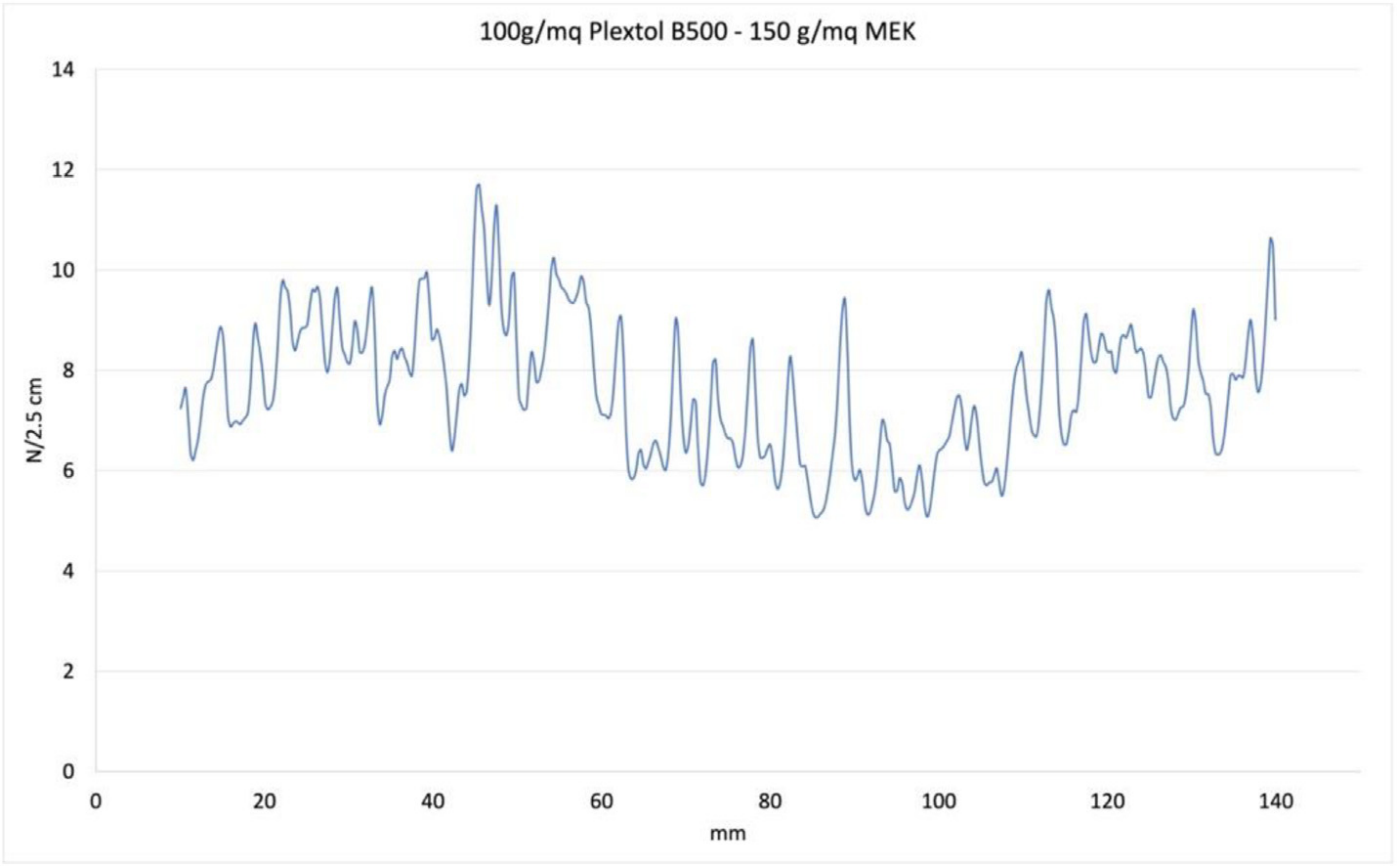
A typical plot for a peel test of a lined canvas sample.

Peel tests are long-stroke uniaxial tensile tests that measure the force required for the mechanical separation of two adherents. The results allow to evaluate the bond strength obtained by a specific bonding technique and to compare it with that obtained by other techniques or their variations. A typical peel test plot is characterized by oscillating values because the curve moves upward until the force buildup is sufficient to cause the bond to fail, which causes a reduction in the reaction force until the movement of the carriages allows new force buildup. Uniaxial testing machines move a single carriage away from a fixed point, but the existing setup allowed us to split the length of the travel between two opposing actuators, making better use of machine space. The sample in Fig. 17 is a lined mockup painting.

## Conclusions

The low-cost biaxial tester is custom-designed and built without the support of a manufacturing company specialized in automation. Easily available components were chosen, and the overall cost is very low (in the range of € 2.5 K at the 2022 prices), if compared with commercial machines (€ 50 K - € 100 K + ) or with other self-built machines, if the cost of a recent comparable example [24] is six times higher. The system was tested to verify error and repeatability, and the machine demonstrated high-performance standards.

The goal of keeping construction not too complex nor expensive was considered crucial because research in painting conservation would benefit from the availability of biaxial tensile testing, and from that of the different mechanical tests the machine can perform. For the same reason, we chose the open-source format to promote the construction and dissemination of similar machines, and to learn from the feedback of potential future users. Software development was challenging as it happened remotely during the pandemic, with the machine's hardware in Italy and the software and firmware being developed in Spain. But if we could do it remotely, the information delivered with this paper would make building a duplicate or a better machine a relatively easy task.

The development of a finely adjustable pre-tensioning procedure was the main unplanned feature during the design of the machine, and its need emerged during early testing. The initial tensile conditions proved to affect the accuracy and repeatability of the tests; therefore, we consider the pre-tensioning procedure an important feature associated with this machine.

## CRediT authorship contribution statement

**Antonio Iaccarino Idelson:** Conceptualization, Methodology, Resources, Investigation, Validation, Funding acquisition, Project administration. **Miguel Sánchez López:** Conceptualization, Methodology, Resources, Software, Validation. **Roger Groves:** Supervision, Writing – review & editing.

## Declaration of Competing Interest

The authors declare that they have no known competing financial interests or personal relationships that could have appeared to influence the work reported in this paper.

## Figures and Tables

**Table:** 

CMD		Parameters	Action
H		-	Prints the command list on the serial port
T		-	Tare load cells
S		-	Stop machine motion
D		-	Toggle ON/OFF load cell measurements printing to the serial port
O		-	Home carriages (innermost position)
I		-	Home carriages (outermost position)
R		-	Set the current location as position 0
?		-	Prints out once the values of the time, elongation and load cells measurements
V		int	Sets Timer1 value in microseconds to determine the step rate. For example, V1000 will cause a speed of 500 steps/second (1/(2*1e-3))=1000/2=500
M		int	Selects the set of motors that will move in the next motion command or test. -1=all, 0..3:invidual, 12,30, 255=none
L		int	Sets the maximum elongation desired for any test. A test will stop moving if that elongation is reached.
X		int	It requests all active axes to start moving at a constant speed at the specified distance in millimeters. If positive, axis motion is outward; otherwise, inward.
C		int	Set the maximum force allowed. The machine stops moving if any load cell exceeds the maximum force (pulling or pushing).
K		float	Set the pretension force for the X axis. This value will be used for the pre-tensioning command only.
@		[float float float float]	With parameters will set the scale for the four load cells. Without parameters, it will print out the current scale factors.
J		float	Set the pretension force for the Y axis. This value will be used for the pre-tensioning command only
W		int	Start the pre-tensioning algorithm for a maximum number of seconds specified by the parameter.
P		-	Start a peel test at the desired speed and elongation
E		-	Print out the status of the end-stop input of each carriage.
A		Int	Set the homing speed (It can be much faster than the test speed).
B		-	Print out the firmware version

## Appendix A

List of the commands

## Footnotes

1 Among others, Instron and Zwick Roell have planar biaxial testing machines displayed on their websites, for applications in general mechanics, elastomer industry, biomechanics.

2 When Antonio Iaccarino Idelson was kindly invited by Christina Young to see the machine.

3 https://www.iso.org/standard/84281.html - last viewed on January 22, 2023. A contact was made with the ISO Technical Committee, but no further information is available.

4 All biaxial tests performed with the machine on different sample sizes produced, so far, comparable or superposable stress–strain plots.

5 The force reaches 75N on each axis for the 25 mm samples.

6 Among others in the literature of this paper, see [4].

10 NEMA 23, with the remarkably high torque value of 4Nm.

11 RM1605 Ballscrew, with Anti-Backlash Ballnut, with C7 class ball bearings.

12 SBR16 supported round rail (16 mm diam shaft, h6 tolerance, induction hardened), 300 mm long, with SBR16 long open linear bearings.

13 S type load cells, class C3.

14 The presence of two opposing load cells on each axis improves the management of sensor-related errors, as readings from two cells are averaged.

15 In this case, TIG welding was used, as it is very controllable and accurate.

16 AkzoNobel Powder Coatings, Interpon 610 NW300M INT 610 HR IRONANTHRACITE.

17 Equilibrarte is a conservation company, with Antonio Iaccarino Idelson as CEO and Technical Director.

18 The high torque value of the motors protects the system with a large margin of safety from the risk that they might lose steps with the loads predicted by the design. In any case, should this ever happen, it would manifest an anomalous behavior in the force/displacement graph, allowing the part of the test following the anomaly to be discarded.

19 HX711 board.

20 All produced in China by XNQJALYCY.

21 Minimum gage length is typically 10.6 mm for the 10 mm sample clamps and 25.8 for the 25 mm clamps.

22 PS2125 produced by Oxford Electrical Products ltd, Oxon UK.

23 Pretensioning time depends on the material’s stiffness and on the accuracy of the clamping procedure.

24 Neoteck, model NTKTL287-FBA.

25 Reference masses were rented from Gibertini Ltd and tests were performed in the presence of qualified technicians from the same company. https://www.gibertini.com/.

## Footnotes

7 Long duration tests (constant elongation or constant load) in the field of paintings conservation have often been adapted to specific needs [10; 11; 15; 16]. Standards are not mentioned in research publications and do not appear to be available.

8 Ibid.

9 Distance is to be divided between the two opposing actuators, thus 175 mm each. ASTM D903-98 prescribes a total run of 304,8 mm.
